# Mass spectrometry for the evaluation of monoclonal proteins in multiple myeloma and related disorders: an International Myeloma Working Group Mass Spectrometry Committee Report

**DOI:** 10.1038/s41408-021-00408-4

**Published:** 2021-02-01

**Authors:** David L. Murray, Noemi Puig, Sigurdur Kristinsson, Saad Z. Usmani, Angela Dispenzieri, Giada Bianchi, Shaji Kumar, Wee Joo Chng, Roman Hajek, Bruno Paiva, Anders Waage, S. Vincent Rajkumar, Brian Durie

**Affiliations:** 1 Department of Laboratory Medicine and Pathology, Mayo Clinic, Rochester, MN USA; 2 Hospital Universitario de Salamanca, Instituto de Investigación Biomédica de Salamanca, Salamanca, Spain; 3 Faculty of Medicine, University of Iceland, Reykjavík, Iceland; 4 Department of Hematologic Oncology & Blood Disorders, Levine Cancer Institute/Atrium Health, Charlotte, NC USA; 5 Department of Hematology, Mayo Clinic, Rochester, MN USA; 6 Division of Hematology, Department of Medicine, Brigham and Women’s Hospital, Harvard Medical School, Boston, MA USA; 7 Cancer Science Institute of Singapore, NUS, Singapore, Singapore; 8 Yong Loo Lin School of Medicine, NUS, Singapore, Singapore; 9 National University Cancer Institute, Singapore, Singapore; 10 Department of Hematooncology, University Hospital Ostrava and Faculty of Medicine, University of Ostrava, Ostrava, Czech Republic; 11 Clinica Universidad de Navarra, Centro de Investigacion Medica Aplicada (CIMA), IDISNA, Pamplona, Spain; 12 Department of Clinical and Molecular Medicine, Norwegian University of Science and Technology, Trondheim, Norway; 13 Department of Hematology, St. Olav’s University Hospital, Trondheim, Norway; 14 Department of Hematology, Cedars-Sinai Outpatient Cancer Center, Los Angeles, CA USA

**Keywords:** Myeloma, Myelodysplastic syndrome

## Abstract

Plasma cell disorders (PCDs) are identified in the clinical lab by detecting the monoclonal immunoglobulin (M-protein) which they produce. Traditionally, serum protein electrophoresis methods have been utilized to detect and isotype M-proteins. Increasing demands to detect low-level disease and new therapeutic monoclonal immunoglobulin treatments have stretched the electrophoretic methods to their analytical limits. Newer techniques based on mass spectrometry (MS) are emerging which have improved clinical and analytical performance. MS is gaining traction into clinical laboratories, and has replaced immunofixation electrophoresis (IFE) in routine practice at one institution. The International Myeloma Working Group (IMWG) Mass Spectrometry Committee reviewed the literature in order to summarize current data and to make recommendations regarding the role of mass spectrometric methods in diagnosing and monitoring patients with myeloma and related disorders. Current literature demonstrates that immune-enrichment of immunoglobulins coupled to intact light chain MALDI-TOF MS has clinical characteristics equivalent in performance to IFE with added benefits of detecting additional risk factors for PCDs, differentiating M-protein from therapeutic antibodies, and is a suitable replacement for IFE for diagnosing and monitoring multiple myeloma and related PCDs. In this paper we discuss the IMWG recommendations for the use of MS in PCDs.

## Background

Plasma cell disorders (PCDs) are a group of diseases characterized by clonal expansion of plasma cells^1^. Central to the diagnosis and monitoring of most PCDs is detection of the monoclonal immunoglobulin components which are generally overproduced by the expanding plasma cell clone. This overproduced monoclonal immunoglobulin (often referred to as an M-protein or paraprotein) typically is an intact immunoglobulin, and also can be either the free light chain (LC) component alone or the heavy chain component alone in rare instances^2^.

While the M-protein is homogeneous and typically constant in any particular patient, the heterogeneity of M-proteins from patient to patient is significant and thus a diverse set of methods are employed to characterize and detect M-proteins^3^. Serum protein electrophoresis (SPEP) enables the detection and relative quantitation of the M-protein, whereas serum immunofixation electrophoresis (IFE) enables establishment of M-protein isotype. Another widely utilized assay is the serum free light chain (sFLC) assay that utilizes specific antibodies for quantitation of circulating free kappa (κ) and lambda (λ) light chains (LCs)^4^. The sFLC assay is an analytically sensitive assay for M-protein detection whereby an abnormal κ/λ FLC ratio (κ/λ < 0.26 or >1.65) suggests the presence of an aberrant plasma or B-cell clone, but not all patients with multiple myeloma (MM) have abnormal sFLC ratios at diagnosis^5^. The sFLC assay has demonstrated a particularly important supportive role in the diagnosis, prognosis, and monitoring of PCDs, especially for amyloidosis and non-secretory MM patients^1,6,7^ In 2009, a panel of members of the IMWG met to develop guidelines for standard investigative workup of patients with suspected MM. The group evaluated studies that compared the diagnostic sensitivity and specificity of different combinations of testing panels and concluded that a combination of SPEP, serum IFE, and sFLC should be used for screening^8^. These protein studies have been incorporated in defining the various PCDs, as well as the uniform response criteria to assess treatment efficacy^9^.

While the clinical utility of the electrophoretic methods to screen and monitor PCDs has been well-established, several changes in the treatment of PCDs are pushing these methods to their analytical limits. Dramatic improvement in the treatment response of MM patients to new chemo and immune therapies is challenging long-held assumptions about this ominous disease. In particular, as durable remissions can now be achieved for most MM patients, highly sensitive methods capable of detecting low-level disease are important for patient counseling. The long-standing routine serum electrophoretic methods (SPEP and IFE) are not capable of providing the analytical sensitivity needed to assess minimal residual disease (MRD). As a result, laboratories have turned to bone marrow (BM) aspirates and/or biopsies and detecting residual malignant plasma cells with high-sensitivity flow cytometry^10^ and their specific VDJH/DJH sequences by next-generation sequencing (NGS)^11^. These BM-based techniques are well-established and currently available for MRD testing after therapy. However, these MRD methods require an invasive procedure and a lab with a higher level of expertise to perform the testing. Attesting to its impact on prognosis, NGS is now an FDA-approved method for measuring MRD in MM. In addition, new monoclonal therapeutic antibodies (t-mAbs) designed to eradicate malignant plasma cells are producing interferences making it difficult to distinguish between a patient’s M-protein and the t-mAb drug on electrophoretic methods^12^.

A search for a more convenient serum-based test to complement BM MRD detection and aid in resolving t-mAb interferences was sought to address limitations in traditional testing. Mass spectrometry (MS) is aptly suited for this task as the improvements in instrumentation and techniques have resulted in increased resolution and sensitivity which have outpaced improvements in electrophoresis.

## M-protein detection by mass spectrometry

The basis of all the mass spectrometric methods for detecting M-proteins is the unique sequence of the antigen binding region, also called the complementarity determining region (CDR) of the Ig^13^. Each plasma cell produces a unique Ig with a specific CDR due to the adaptive immune system’s optimization of the CDR via somatic hypermutation to increase the affinity of the Ig to its target antigen. The resulting CDR amino acid sequence is unique, and each plasma cell clone has a different peptide sequence and overall mass, which is the basis of the M-protein detection by MS^14^. Two MS methods have emerged in the literature. Both methods start with immune-enrichment of patient immunoglobulins (Igs) but differ on the analytical target used to detect the M-protein. One method utilizes Ig trypsin digestion and detection of peptides specific to the M-protein CDR^15–18^. This method has been termed the “clonotypic peptide” approach. The second method utilizes total LC mass distributions from Igs which have been chemically reduced and denatured into heavy and light chain components^19,20^. This method will be termed intact LC mass measurements herein.

### The clonotypic peptide approach

The clonotypic peptide approach has been employed to monitor MM patients^15^, assess for MRD in complete response (CR) patients^16,21^, and to differentiate M-proteins from Daratumumab^17^. The clonotypic method is very analytically sensitive with M-protein detection rates down to 0.001 g/L giving it potential to be a serum-based MRD method^16^. However, the method has some limitations that hinder it from being suitable for all patients. In some patients with M-proteins above 10 g/L, only constant/framework peptides were detected making it impossible to follow or detect M-proteins in these patients. In addition, as the CDR also contains framework regions, which have less diversity across the polyclonal background, clonotypic peptide selection needs to be done judiciously to assure the uniqueness of the clonotypic peptide to the M-protein. As such, the success of identifying a clonotypic peptide still requires genetic CDR sequence information from the malignant plasma cells. Once a potential clonotypic peptide is determined, a search of the human proteome is necessary to assure its uniqueness. Unfortunately, a complete library of the possible human CDR tryptic peptides is not yet available. All this requires very thorough understanding of immunoglobulin CDR region to assure definitive test results. Lastly, screening for M-proteins using the clonotypic peptide method has not been demonstrated. Recent work utilizing IFE gels followed by the clonotypic approach has aided in simplifying the pre-analytical work for the clonotypic method^22^, but does not remove IFE from its screening role.

More clinical data is still needed to determine if the MRD positivity by the clonotypic approach translates into disease-free and overall survival benefits. While most studies utilizing the clonotypic method detect the presence of the M-protein in samples from patients who are BM MRD-negative, the clinical implications of this will require more time to elucidate.

### Intact Ig light chain approach

A second more simpler and practical approach for M-protein detection relies on scanning the overall mass distribution of denatured intact Ig LCs. In this method, the polyclonal Ig LC mass distribution results in two normal distributions, one for the lambda and second for the kappa LCs^23^. The first version of this intact LC mass measurement method was performed on higher performance mass spectrometers (ESI-Q-TOFs) with liquid chromatography. This method (termed monoclonal immunoglobulin rapid accurate mass measurements or miRAMM) was found to have an analytical limit of quantitation of 0.05 g/L and a limit of detection of 0.01 g/L. Later this method was adapted to matrix-assisted laser desorption/ionization-time-of-flight (MALDI-TOF) mass spectrometry (MS)^24^, which eliminated the chromatography step and reduced analytical time from 20 min to 10 s. Eventually, the MALDI-TOF method was modified to include immuno-enrichments to conclusively identify the M-protein isotype in a similar fashion to IFE^20,25,26^. The discussion and recommendations for MS contained in this working group report, unless otherwise specified, pertain to this intact LC MALDI-TOF MS method.

The intact LC MALDI-TOF MS assay is referred to as Mass-Fix at the Mayo Clinic to emphasize its similarity to conventional immunofixation. It has been clinically validated as a lab-developed test at the Mayo Clinic^27^. At this point in time, over 40,000 clinical samples have been analyzed by the intact LC MALDI-TOF method at Mayo Clinic. The Mass-Fix assay has been found to be comparable to IFE in terms of assay turnaround time and ease of interpretation with the added benefit of reduced labor. These features make the intact LC method more economically attractive in comparison to the clonotypic method. Commercial efforts are being made to automate the technique and provide a high-throughput method which would be widely accessible for routine laboratory implementation. Validation is currently being performed in several clinical laboratories.

#### Analytical and clinical performance of intact LC MALDI-TOF assay compared to IFE

Several comparisons of the intact LC MALDI-TOF MS and IFE have been published^20,21,25^. The ability of intact LC MALDI-TOF MS method to detect M-proteins was retrospectively evaluated on a large cohort of patient samples with physician-ordered SPEP and IFE, which included a wide variety of M-proteins encountered in clinical practice. MALDI-TOF MS detected M-proteins in 100% of samples that were positive by both SPEP and IFE (*n* = 84) and in 97% of samples positive by IFE but negative by SPEP (68 of 70). In samples negative by both SPEP and IFE (*n* = 28), MALDI-TOF identified one positive case^20^. To test analytical sensitivity, a cohort of 27 PCD patients was diluted into normal human serum and both IFE and intact LC MALDI-TOF were performed. The percentage of M-proteins detected by the intact LC MALDI-TOF was significantly higher than IFE at every dilution. A second prospective study involving 290 patients with documented PCDs demonstrated that substitution of serum and urine intact LC MALDI-TOF testing results for convention IFE results allowed for detection of disease in 18 more patients. Overall, MS had higher clinical sensitivity than IFE in detecting M-proteins in patients with abnormal FLC ratios. A combination of FLC, serum and urine Mass-Fix performed equally as well as current IMWG recommendations of PEL, IFE, and sFLC^28^. Incorporation of serum FLC MALDI-TOF MS^26^ has been shown to improve the sensitivity of M-protein detection during monitoring of AL amyloidosis^29,30^. The intact LC method has also been used for the diagnosis of heavy chain diseases^31^.

A more recent study confirmed the increased sensitivity and accuracy of intact LC MALDI-TOF MS over IFE. In this study, 226 patients from the Olmsted screening cohort^32^ who were initially negative for a PCD by SPEP but who were found to have a PCD on later follow-up were studied. Since the initial screen was performed by SPEP, the study assessed the ability of IFE, the intact LC MALDI-TOF method (Mass-Fix), as well as a more sensitive miRAMM method to detect the M-protein in the original sample. M-protein would have been detected in 11%, 50%, and 67% of these participants if initial screening was performed by IFE, MALDI-TOF, and miRAMM, respectively^33^. A preliminary study of the pre-commercial intact LC MALDI-TOF method (QIP-MS, The Binding Site) utilizing samples from the GEM-CESAT trial patients demonstrated moderate correlation with MRD assessment by next-generation flow MRD status^34^.

The intact LC method MS can give useful information to discriminate between t-mAbs and M-proteins as the mass of the t-mAb LC can be measured. Using the intact LC MALDI-TOF method, daratumumab and IgG kappa M-proteins were distinguishable in 84% of the samples tested^35^. The inability to discriminate between daratumumab and M-protein was most likely due to the lower resolution of the MALDI-TOF assay, as studies involving the higher resolution LC-ESI-QTOF (miRAMM) MS had a 100% success rate of differentiating the t-mAb (daratumumab, elotuzumab, and isatuximab) from M-protein^36^.

A previously underappreciated finding of M-proteins when employing the intact LC method was the observation of M-proteins with a LC mass that was broad and outside the normal mass ranges of kappa and lambda LCs^25,28^, Additional studies demonstrated that these LCs contained N-linked glycosylation and were to be more prevalent in patients with AL-amyloidosis^37^ and cold agglutinin disease^38^ than other PCDs. Interestingly, LC glycosylation was also found to be an independent risk factor for the progression of MGUS to myeloma and related disorders^39^. These glycosylated LCs were demonstrated to be present years before the diagnosis of myeloma of related disorder and hence may allow for identification of patients at higher risk for PCDs^40^.

MS using the more sensitive miRAMM intact LC approach may have a role in MRD detection. In one study, residual monoclonal proteins were detected in patients who were in stringent CR (sCR) (10^−4^ to 10^−5^ by 6 color flow cytometry) in 80% (*n* = 16) and 60% (*n* = 25) of patients at 6–12 months, respectively^41^. The influence of Ig half-life on the MRD data needs to be further elucidated. However, since miRAMM MS is a serum-based assay, the test could be performed serially allowing the rate of response to be assessed over time. A second more recent study also compared the intact LC MALDI-TOF MS with BM flow cytometry-based MRD^42^. This exploratory study demonstrated MALDI‐TOF MS was concordant with MRD flow for 44/71 (62%) patients (*p* = 0.342). Amongst the 27 discordant cases, 17 were detectable only by MALDI-TOF MS, and 10 detectable only by MRD flow. Additional prospective studies are required to delineate the role and timing of these modalities in assessing MRD.

#### Recommendations

We conclude that MS has the advantage of increased accuracy, documented clinical and analytic sensitivity, and the intact LC MALDI-TOF method is easier on laboratory work flow for the detection of M-proteins^20^. As summarized in Table 1, the IMWG Mass Spectrometry Committee endorses detection of M-proteins by MS (intact MALDI-TOF method) as an alternative to IFE for clinical practice and clinical trials. The group also endorses MS for distinguishing residual M-protein from therapeutic monoclonal antibodies for clinical practice, and for accurate interpretation and determination of complete response in clinical trials. We recognize that using mass spectrometric methods instead of conventional IFE may lead to lower rates of CR, and therefore cross-comparisons of CR rates in trials done in different time periods is not recommended. We hope, with further data, that mass spectrometric methods (MALDI-TOF, miRAMM, or clonotypic peptide approach) may provide the ability to test for measurable disease in the peripheral blood and help guide timing of BM tests for next-generation flow cytometry and NGS studies. Prospective validation of the impact of MRD negativity based on MS on patient progression-free survival and overall survival in relationship with BM NGS MRD is strongly encouraged as part of clinical trials. Finally, we encourage further investigations to further clarify the relationship and implications of N-linked glycosylation and AL amyloidosis.

**Table 1:** List of IMWG recommendations regarding mass spectrometry.

Intact LC MALDI-TOF can be used in lieu of immunofixation in the clinically assessment of patients and the assessment of patients on clinical trials.
We endorse the use of mass spectrometry to aid in distinguishing therapeutic antibodies from endogenous M-proteins.
We recognize that using mass spectrometric methods in lieu of conventional IFE may lead to lower rates of CR, and therefore cross-comparisons of CR rates in trials done in different time periods is not recommended.
We endorse the collection of further data from mass spectrometry (MALDI-TOF, miRAMM, or clonotypic peptide approach) to document the ability to test for MRD negativity in the peripheral blood, and to guide timing of BM tests for next-generation flow cytometry and NGS studies.
We encourage further investigations to further clarify the relationship and implications of N-linked glycosylation in MGUS progression to myeloma and AL amyloidosis.

## Supplementary information

AJR Checklist.
